# Subcutaneous Four-Week Repeated Dose Toxicity Studies of Rice Cell-Derived Recombinant Human Granulocyte-Macrophage Colony Stimulating Factor in Rats

**DOI:** 10.5487/TR.2008.24.4.315

**Published:** 2008-12-01

**Authors:** Jung Eun Ji, Jung Min Lee, Jong Min Choi, Young Hwa Choi, Eun Kyung Kim, So Jung Chu, Seok Kyun Kim, Kyong Hoon Ahn, Dong Hoon Lee, Ha Hyung Kim, Kyuboem Han, Dae Kyong Kim

**Affiliations:** 110 Department of Environmental & Health Chemistry, Physical Pharmacy Laboratory, College of Pharmacy, Chung-Ang University, Seoul, 156-756 Korea; 210 Department of Environmental & Health Chemistry, Hanson Biotech Co., Ltd., Daejeon, 305-811 Korea

**Keywords:** Recombinant hGM-CSF, Rice cells, Repeated dose toxicity, SD rats

## Abstract

Recombinant human granulocyte-macrophage colony stimulating factor (hGM-CSF) is a glycoprotein and hematopoietic growth factors that regulates the proliferation of myeloid precursor cells and activates mature granulocytes and macrophages. In a previous study, we reported that hGM-CSF could be produced in transgenic rice cell suspension culture, termed rhGM-CSF. In the present study, we examined the repeated dose toxicity of rhGM-CSF in SD rats. The repeated dose toxicity study was performed at each dose of 50 and 200 µg/kg subcutaneous administration of rhGM-CSF everyday for 28-days period. The results did not show any changes in food and water intake. There were also no significant changes in both body and organ weights between the control and the tested groups. The hematological and blood biochemical parameters were statistically not different in all groups. These results suggest that rhGM-CSF may show no repeated dose toxicity in SD rats under the conditions.

## INTRODUCTION

Human granulocyte-macrophage colony-stimulating factor (hGM-CSF) was the first CSF to be purified, cloned and expressed using recombinant DNA technology (Armitage, 1998). The mature GM-CSF is a polypeptide of 127 amino acid residues (Cantrell *et al.*, 1985; Lee *et al.*, 1985; Wong *et al.*, 1985). It regulates the production and the function of white blood cells including granulocytes and monocytes, which are essential in fighting infection (Metcalf, 1991). Due to its important role in the immune system, hGM-CSF has an increasing clinical applications in the treatment of neutropenia, aplastic anemia and oral mucositis commonly caused by cancer chemotherapy or radiotherapy (Vazquez, 1998). Additionally it greatly reduced the risk of infection which of associated with bone marrow transplantation via accelerating the response of neutrophils (Rasko and Goygh, 1994).

cDNA of hGM-CSF has been cloned and used to produce hGM-CSF in *Escherichia coli*, *Saccharomyces cerevisiae,* and Chinese hamster ovarian (CHO) cells, which are termed molgramostim, sargramostim and regramostim, respectively (Armitage, 1998). The majority of therapeutic recombinant proteins are produced in mammalian cells or single cell organisms such as bac-

Abbreviations: Recombinant human granulocyte-macrophage colony stimulating factor (hGM-CSF), 50% lethal dose ($LD_{50}$), *Escherichia coli* (*E. coli*), Chinese hamster ovary (CHO), rice cells-derived hGM-CSF (rhGM-CSF), phosphate buffered saline (PBS), ethylenediaminetetraacetic acid (EDTA), red blood cell (RBC), white blood cell (WBC), neutrophil (NEU), lymphocyte (LYM), monocyte (MONO), eosinophil (EOS), basophil (BASO), platelet (PLT), hematocrit (HCT), mean corpuscular volume (MCV), hemoglobin (HGB), mean corpuscular hemoglobin (MCH), mean corpuscular hemoglobin concentration (MCHC), aspartate aminotransferase (AST), alanine aminotransferase (ALT), alkaline phosphatase (ALP), blood urea nitrogen (BUN), creatinine (CRE), glucose (GLU), total cholesterol (CHO), total protein (PRO), creatine phosphokinase (CPK), albumin (ALB), total bilirubin (BIL), yeast-derived hGM-CSF (yhGM-CSF), subcutaneous (*s.c.*), body weight (b.wt.), standard deviation (SD).

teria and yeast. However, these animal and microbial expression systems are prone to potential viral contamination. To achieve greater cost savings and to address a capacity deficit in the global demand for recombinant protein-based pharmaceuticals, green plants are being explored as alternative protein production hosts (Giddings, 2003) and higher plants have provided a safe and economical way of producing plant-made pharmaceuticals (PMP) (Sardana *et al.*, 2007).

Different plant tissue such as leaves, seeds, and tubers have been engineered for producing useful recombinant proteins (Vandekerckhove *et al.*, 1989). In a number of studies, plants such as tobacco have been used as a host, but these non-edible plants have some major drawbacks (Shin *et al.*, 2007). On the opposite end of the biochemical spectrum is the medically approved and nutritionally enviable rice flour (Sardana *et al.*, 2007).

In recent study, we found that the glycosylation pattern of rice cell-derived hGM-CSF, termed rhGM-CSF, was different from that of yeast-derived hGM-CSF, termed yrGM-CSF, based on analytical investigation for the purified recombinant protein obtained from rice cells. In particular, rhGM-CSF is more heavily glycosylated than yhGM-CSF or CHO cell-derived one, termed chGM-CSF (Kim *et al.*, 2008).

The products differ in the specific amino acid sequences and degree of glycosylation for instance *E. coli*-derived molgramostim has a molecular weight of 14.5 kDa and is non-glycosylated, whereas yhGM-CSF *O*-glycosylated has a molecular weight of 16~18 kDa.

The latter one contains leucine instead of proline at position 23 of mature form of wild-type hGM-CSF (Okamato *et al.*, 1991; Dorr, 1993).

The degree of glycosylation may have important clinical implications, since it might affect antigenicity, toxicity and pharmacokinetics (Lieschke and Burgess, 1992; Hovgaard *et al.*, 1993; Ragnhammar *et al.*, 1994; Armitage, 1998; Wadhwa *et al.*, 1999). It was known that the degree of glycosylation varies according to cell type and the *in vivo* stability increase with *N*-glycan content (Okamato *et al.*, 1991). These results suggest that the glycans on rhGM-CSF are superior to those on yhGM-CSF in terms of *in vivo* stability.

Therefore, the culture systems of plant cells may be the most favorable means of producing small-to-medium quantities of high-price, high-purity, specialty of recombinant proteins, despite the fact that these systems are relatively new (Doran, 2000). Although it is generally accepted that the culture systems of plant cells have some advantages compared to those of animal cells, it is pivotal to evaluate whether rhGM-CSF reveals a toxic effect due to a different pattern of glycosylation or an unknown factor. Recently, we found that rhGM-CSF did not show any toxicity in intravenous single and two weeks repeated dose (Ji *et al.*, 2007).

In the present study, we examined subcutaneous (*s.c.*) 28-days repeated dose toxicity of rhGM-CSF produced by rice cell suspension culture.

## MATERIALS AND METHODS

***Drugs and chemicals.*** rhGM-CSF was produced as described previously (Shin *et al.*, 2003) and purified to near homogeneity of < 95% purity from the cell culture media (Han *et al.*, unpublished data). The amount of the protein was measured by Lowry method (Lowry *et al.*, 1951) and dissolved in phosphate buffered saline (PBS). All chemicals were kept under sterilized condition.

***Experimental animals.*** For the repeated dose toxicity study, SD rats weighting 90 ± 5 g (female), 95 ± 5 g (male) (4 weeks-old) were purchased from Japan SLC, Inc. (Shizuoka, Japan). The animals were housed under standard conditions of a good laboratory practice (GLP)-based facility and kept in a well-ventilated and specific pathogen-free room (12 hr light and 12 hr darkness and room temperature of 25 ± 2$^{o}$C). All the animals were allowed sterilized tap water and commercial rodent chow (Japan SLC, Shizuoka, Japan) ad libitum. The study was approved from the department's ethical committee for the use of the animals and the study design. Sterile solution of rhGM-CSF in saline was administrated subcutaneously at the indicated doses to treated groups, where as the control received 0.5 ml vehicle. The animals were observed continuously during the test period for any signs of behavioral changes, toxicity and mortality.

***Repeated dose toxicity study.*** 18 male and 18 female SD rats were divided into three groups according to the dose, respectively, and were subcutaneously administrated for 28 days daily. The treated groups were administrated at the doses of 50 and 200 μg/kg b.wt. and the control groups received 0.5 ml of the vehicle alone. The highest dosage level was 200 μg/kg/day according to pharmacology and toxicology studies of the product of yhGM-CSF (Leukine®; Bayer HealthCare Pharmaceuticals Inc., Wayne, NJ) (Krumwieh *et al.*, 1990). Food and water intake, body weight, and general behavior were observed daily. All animals were sacrificed at 28$^{th}$ day and the organs of each rat were isolated for examining the morphological changes and weights. Blood samples were withdrawn in EDTA tubes to obtain plasma for hematological and biochemical assays. Plasma samples were stored at -70°C pending assay.

***Hematological assay.*** The blood samples collected in EDTA-anti-coagulated tubes was analyzed for hematological assay using an automatic hematological assay analyzer (ADVIA 2120, SIEMENS). Different tested hematological parameters were as follows: red blood cell (RBC), white blood cell (WBC), neutrophil (NEU), lymphocyte (LYM), monocyte (MONO), eosinophil (EOS), basophil (BASO), platelet (PLT), hematocrit (HCT), mean corpuscular volume (MCV), hemoglobin (HGB), mean corpuscular hemoglobin (MCH), and mean corpuscular hemoglobin concentration (MCHC).

***Biochemical assay.*** The levels of aspartate aminotransferase (AST), alanine aminotransferase (ALT), alkaline phosphatase (ALP), blood urea nitrogen (BUN), creatinine (CRE), glucose (GLU), total cholesterol (CHO), total protein (PRO), creatine phosphokinase (CPK), albumin (ALB), total bilirubin (BIL) were determined using an Technicon RA-XT auto-analyzer (Technicon Corp., New York, USA) (Han *et al.*, 2004; Kim *et al.*, 2007).

***Statistical analyses.*** The values are expressed as mean ± standard deviation (SD). Statistical analysis was performed with ANOVA (SPSSD 12.0, Chicago, USA). A *P* value of less than 0.05 was considered statistically significant.

## RESULTS AND DISCUSSION

In a previous study, we examined the effect of single intravenous dose of rhGM-CSF on rat SD. We found that there were no mortality or any signs of behavioral changes or toxicity up to the dose level of 1,000 μg/kg b.wt. of rhGM-CSF (Ji *et al.*, 2007). Thus, the lethal dose ($LD_{50}$) was not found.

Another product of yhGM-CSF, Leucogen®, was also known to show a protective effect against ulcerative mucositis in hamster buccal pouch (Cho *et al.*, 2006). They reported that the topographical administration of ~2.5 μg/kg b.wt. (100 ± 20 g) of the low dose of the yhGM-CSF showed a good healing effect against ulcerative mucositis. Thus, the dose of 1,000 μg/kg b.wt. in the rats could be higher by ~40-fold than the effective one in hamster. The results suggest that considering the efficacy of yhGM-CSF being currently used for cancer patients, rhGM-CSF may be non-toxic in both male and female rats (Ji *et al.*, 2007).

Recently, we also found that the repeated *i.v.* dose toxicity study of rhGM-CSF for 14-days period at the doses of 50 and 200 μg/kg did not show any signs of toxicity in SD rats (Ji *et al.*, 2007). In this study, the rats were subcutaneously administrated with hGM-CSF for 28 days. The effects of rhGM-CSF on body and organ weights in both male and female rats were determined, respectively. The weights in the treated male and female rats were slightly decreased at the $7^{th}$, $14^{th}$, $21^{th}$ and $28^{th}$ days (Table 1). However, no significant differences existed in the absolute and relative weights of all isolated organs in both the treated and control rats except for the significant and dose-dependent increase ($p < 0.05$) in spleen and lung weights in the male Group II and III, compared to the control rats (Table 2). Furthermore, no lethality was recorded for any dose during the experimental period. Moreover, we examined the difference in food consumption and water between the treated and control rats. As shown in Table 3, there was no significant difference in food and water intake in both male and female rats.

Afterwards, the influences of rhGM-CSF on hematological and biochemical parameters were determined. First, hematological parameters and their comparisons among all groups are presented in Table 4 and 5. The

**Table 1.** Changes in body weights (g) of rats treated with various doses of rhGM-CSF

| Sex | Group | Days | | | | |
|---|---|---|---|---|---|---|
| | | 0 | 7 | 14 | 21 | 28 |
| Male | Group I | 94.3 ± 2.6 | 151.5 ± 10.2 | 197.7 ± 15.8 | 256.8 ± 19.2 | 286.3 ± 19.1 |
| | Group II | 95.0 ± 6.8 | 153.2 ± 11.6 | 202.0 ± 13.9 | 261.7 ± 16.4 | 293.8 ± 17.1 |
| | Group III | 97.5 ± 3.6 | 159.7 ± 6.3 | 211.8 ± 7.7 | 270.3 ± 12.0 | 292.7 ± 8.7 |
| Female | Group IV | 92.5 ± 4.9 | 129.7 ± 4.4 | 156.3 ± 4.3 | 179.8 ± 3.5 | 200.2 ± 6.1 |
| | Group V | 89.8 ± 5.1 | 128.8 ± 7.2 | 157.8 ± 7.4 | 185.2 ± 10.2 | 205.0 ± 16.0 |
| | Group VI | 88.2 ± 5.2 | 131.8 ± 5.3 | 157.8 ± 7.8 | 179.3 ± 11.1 | 200.7 ± 12.6 |

Results were expressed as mean ± SD (n = 6). Control was treated with the same volume of PBS. Group I and IV were control; Group II and V were treated with rhGM-CSF 50 μg/kg b.wt., and Group III and VI were treated with rhGM-CSF 200 μg/kg b.wt. Body weights (g) were estimated at 0, 7, 14, 21, 28th day following treatment.

**Table 2.** Change in organ weights (g) of rats treated with various doses of rhGM-CSF

| Sex | | Group | Organ | | | | | |
|---|---|---|---|---|---|---|---|---|
| | | | Liver | Spleen | Heart | Kidney | Lung | Testis |
| Male | Absolute | Group I | 12.66 ± 1.91 | 0.59 ± 0.09 | 1.11 ± 0.12 | 1.32 ± 0.20 | 1.42 ± 0.12 | 1.64 ± 0.13 |
| | | Group II | 12.24 ± 1.73 | 0.65 ± 0.07 | 1.14 ± 0.19 | 1.23 ± 0.15 | 1.61 ± 0.10 | 1.59 ± 0.10 |
| | | Group III | 11.27 ± 1.09 | 0.69 ± 0.05 | 1.13 ± 0.05 | 1.38 ± 0.15 | 1.69 ± 0.07 | 1.65 ± 0.17 |
| | Relative | Group I | 44.29 ± 6.48 | 2.05 ± 0.25 | 3.88 ± 0.40 | 4.62 ± 0.79 | 4.95 ± 0.39 | 5.75 ± 0.55 |
| | | Group II | 41.49 ± 3.63 | 2.20 ± 0.19* | 3.86 ± 0.46 | 4.16 ± 0.33 | 5.48 ± 0.31* | 5.41 ± 0.39 |
| | | Group III | 38.51 ± 3.44 | 2.37 ± 0.14* | 3.87 ± 0.13 | 4.72 ± 0.48 | 5.76 ± 0.20* | 5.64 ± 0.49 |
| Female | Absolute | Group IV | 7.82 ± 1.53 | 0.45 ± 0.08 | 0.77 ± 0.02 | 0.84 ± 0.07 | 1.27 ± 0.08 | nil |
| | | Group V | 8.48 ± 1.60 | 0.51 ± 0.09 | 0.8 ± 0.09 | 0.85 ± 0.05 | 1.26 ± 0.09 | nil |
| | | Group VI | 8.27 ± 1.87 | 0.49 ± 0.10 | 0.78 ± 0.07 | 0.87 ± 0.07 | 1.31 ± 0.15 | nil |
| | Relative | Group IV | 39.09 ± 7.80 | 2.22 ± 0.38 | 3.83 ± 0.15 | 4.18 ± 0.36 | 6.34 ± 0.44 | nil |
| | | Group V | 41.35 ± 6.90 | 2.49 ± 0.37 | 3.90 ± 0.28 | 4.15 ± 0.23 | 6.19 ± 0.67 | nil |
| | | Group VI | 40.95 ± 7.49 | 2.44 ± 0.38 | 3.90 ± 0.16 | 4.33 ± 0.13 | 6.53 ± 0.41 | nil |

Results were expressed as mean ± SD (n = 6). Each organ was extracted and weighed after blood sampling when the final injection finished. The absolute organ weight (g) was measured and then relative organ weight (mg/g) was calculated for the following organs of all experimental animals when they were sacrificed. Group I and IV were controls, Group II and V were treated with rhGM-CSF 50 μg/kg b.wt., and Group III and VI were treated with rhGM-CSF 200 μg/kg b.wt. Rats were sacrificed at 29th day.
*$p < 0.05$ compared to that of vehicle control by ANOVA test.

**Table 3.** Change in food and water consumptions of rats treated with rhGM-CSF

| | Group I | Group II | Group III | Group IV | Group V | Group VI |
|---|---|---|---|---|---|---|
| | Male | | | Female | | |
| Food | 33.26 ± 2.85 | 32.56 ± 3.08 | 32.99 ± 2.73 | 31.49 ± 5.12 | 30.28 ± 4.19 | 31.36 ± 3.95 |
| Water | 62.56 ± 4.34 | 62.94 ± 5.61 | 63.75 ± 7.00 | 62.19 ± 2.01 | 61.86 ± 2.10 | 61.64 ± 1.73 |

Results were expressed as mean ± SD (n = 6). Each value represents weight (g/rat/day) and volume (ml/rat/day) of average consumptions of food and water. Group I and IV were controls, Group II and V were treated with rhGM-CSF 50 μg/kg b.wt., and Group III and VI were treated with rhGM-CSF 200 μg/kg b.wt. Rats were sacrificed at 29th day.

**Table 4.** Effects of rhGM-CSF on hematological parameters

| Sex | Group | HGB g/dl | HCT % | MCV fL | MCH pg | MCHC g/dl |
|---|---|---|---|---|---|---|
| Male | Group I | 13.0 ± 2.60 | 40.5 ± 0.85 | 55.3 ± 2.39 | 19.3 ± 0.42 | 34.9 ± 0.95 |
| | Group II | 14.0 ± 1.22 | 42.0 ± 1.83 | 55.3 ± 1.32 | 19.7 ± 0.35 | 35.6 ± 0.20 |
| | Group III | 14.6 ± 0.95 | 41.6 ± 2.77 | 54.8 ± 2.66 | 19.2 ± 0.65 | 35.1 ± 0.76 |
| Female | Group IV | 14.6 ± 1.30 | 41.6 ± 3.31 | 53.8 ± 1.08 | 18.9 ± 0.25 | 35.0 ± 0.46 |
| | Group V | 14.2 ± 1.23 | 41.2 ± 3.23 | 55.3 ± 0.74 | 19.0 ± 0.50 | 34.4 ± 0.45 |
| | Group VI | 14.2 ± 1.01 | 40.8 ± 1.87 | 54.7 ± 1.18 | 19.0 ± 0.96 | 34.6 ± 1.05 |

Results were expressed as mean ± SD (n = 6). Group I and IV were controls, Group II and V were treated with rhGM-CSF 50 μg/kg b.wt., and Group III and VI were treated with rhGMCSF 200 μg/kg b.wt. Hematological parameters were measured within 4 hr after blood sampling.

results show that there is no differences between control and treated rats in all the parameters in both male and female rats, suggesting that rhGM-CSF may be hematologically safe even if 200 μg/kg b.wt. was subcutaneously injected daily for 28 days. This dose could be higher by ~8-fold than the single effective dose in hamster (Ji *et al.*, 2007). On the other hand, the counts of blood cells were examined after rhGM-CSF was subcutaneously injected daily for 28 days. As shown in Table 5, the numbers of all types of blood cells measured were not significantly influenced ($p < 0.05$) by the *s.c.* injection of rhGM-CSF, further supporting that the treatment of rhGM-CSF of 200 μg/kg b.wt. for 28 days daily might be hematologically safe in both male and female rats.

Treatment with rhGM-CSF did not significantly affect the level of AST and ALT (Table 6). A significant and dose-dependent increase ($p < 0.05$) in ALP level was observed in female group V and VI, compared to the control rats, but not in the male Group III. In case of T-BIL, its level in female rats slightly decreased.

In our recent study, we found that the glycosylation

**Table 5.** Effect of rhGM-CSF on hematological parameters

| Sex | Group | PLT $10^3/mm^3$ | RBC $10^6/mm^3$ | WBC $10^3/mm^3$ | NEU $10^3/mm^3$ | LYM $10^3/mm^3$ | MONO $10^3/mm^3$ | EOS $10^3/mm^3$ | BASO $10^3/mm^3$ |
|---|---|---|---|---|---|---|---|---|---|
| Male | Group I | 1186.7 ± 34.65 | 6.67 ± 1.43 | 7.54 ± 0.75 | 0.67 ± 0.34 | 4.81 ± 2.34 | 0.26 ± 0.11 | 0.06 ± 0.03 | 0.07 ± 0.06 |
| | Group II | 1210.0 ± 199.09 | 6.41 ± 1.84 | 6.06 ± 1.75 | 0.52 ± 0.36 | 3.94 ± 2.67 | 0.13 ± 0.10 | 0.04 ± 0.02 | 0.10 ± 0.07 |
| | Group III | 1149.5 ± 130.90 | 7.58 ± 0.34 | 6.49 ± 1.28 | 0.42 ± 0.07 | 3.29 ± 1.78 | 0.13 ± 0.06 | 0.07 ± 0.01 | 0.07 ± 0.04 |
| Female | Group IV | 1151.0 ± 67.18 | 7.72 ± 0.65 | 6.49 ± 0.40 | 0.62 ± 0.37 | 4.40 ± 1.39 | 0.26 ± 0.10 | 0.14 ± 0.09 | 0.07 ± 0.05 |
| | Group V | 1163.0 ± 78.73 | 7.45 ± 0.51 | 5.24 ± 0.30 | 0.54 ± 0.11 | 4.36 ± 0.32 | 0.17 ± 0.08 | 0.09 ± 0.01 | 0.08 ± 0.03 |
| | Group VI | 1136.0 ± 138.18 | 7.47 ± 0.33 | 5.97 ± 0.45 | 0.85 ± 0.31 | 4.53 ± 1.47 | 0.19 ± 0.08 | 0.12 ± 0.04 | 0.14 ± 0.06 |

Each result was expressed as mean ± SD (n = 6). Group I and IV were controls, Group II and V were treated with rhGM-CSF 50 μg/kg b.wt., and Group III and VI were treated with rhGMCSF 200 μg/kg b.wt. Hematological parameters were measured within 4 hr after blood sampling.

**Table 6.** Effects of rhGM-CSF on biochemical parameters

| Sex | Group | AST IU/L | ALT IU/L | ALP IU/L | T-BIL mg/dl |
|---|---|---|---|---|---|
| Male | Group I | 70.20 ± 6.46 | 45.40 ± 8.19 | 371.20 ± 39.16 | 0.33 ± 0.10 |
| | Group II | 64.12 ± 5.03 | 41.28 ± 4.81 | 321.37 ± 39.99 | 0.26 ± 0.06 |
| | Group III | 74.87 ± 9.87 | 50.20 ± 9.04 | 359.33 ± 41.22 | 0.39 ± 0.33 |
| Female | Group IV | 80.02 ± 11.19 | 46.03 ± 8.82 | 259.93 ± 19.23 | 0.36 ± 0.17 |
| | Group V | 74.60 ± 5.04 | 42.67 ± 5.38 | 304.68 ± 32.23* | 0.35 ± 0.20 |
| | Group VI | 87.40 ± 12.21 | 46.75 ± 7.43 | 337.18 ± 28.86* | 0.30 ± 0.13 |

Results were expressed as mean ± SD (n = 6). Group I and IV were control, Group II and V were treated with rhGM-CSF 50 μg/kg b.wt., and Group III and VI were treated with rhGM-CSF 200 μg/kg b.wt. Rats were sacrificed at 29th day.

pattern of rhGM-CSF was different from that of yhGM-CSF, based on analytical investigation for the purified recombinant protein obtained from rice cells. In particular, rhGM-CSF is more heavily glycosylated than yhGM-CSF or CHO cells-derived one (Kim *et al.*, 2008). This results suggest that rhGM-CSF might be more hydrophilic and retainable to the mucus of buccal pouches, and in turn could be more effective in ulcerative mucositis of cancer patients induced by a chemotherapeutic agent than yhGM-CSF or CHO cells-derived one (Ji *et al.*, 2007). Furthermore, the heavier glycosylation of rhGM-CSF may render its blood half-life to be longer. Our recent results indicated that rhGM-CSF remains longer in the serum with a higher level compared to yhGM-CSF (Kim *et al.*, 2008).

As described previously, biologically active recombinant hGM-CSF, a protein biological with many applications in medicine and research, has been specifically produced in the seeds of transgenic rice plants at high levels. As rice is primarily a self-pollinated crop (Song *et al.*, 2003), it offers particular attraction in terms of containment of the transgenes, in addition to providing obvious advantages associated with producing protein-based medicines in seeds for direct administration to human patients (novel drug delivery).

In summary, there were no significant changes in both body and organ weights between control and test groups of SD rats when rhGM-CSF was daily administered via a *s.c.* route for four weeks. The hematological and blood biochemical parameters were statistically not significant in all groups.

## ACKNOWLEDGEMENTS

This research was supported by a grant for the next generation from Ministry of Knowledge Economy of Korea and by the Seoul R&BD Program (10550).

## Abbreviations

hGM-CSF: Recombinant human granulocyte-macrophage colony stimulating factor
LD_50_: 50% lethal dose
E. coli: Escherichia coli
CHO: Chinese hamster ovary
rhGM-CSF: rice cells-derived hGM-CSF
PBS: phosphate buffered saline
EDTA: ethylenediaminetetraacetic acid
RBC: red blood cell
WBC: white blood cell
NEU: neutrophil
LYM: lymphocyte
MONO: monocyte
EOS: eosinophil
BASO: basophil
PLT: platelet
HCT: hematocrit
MCV: mean corpuscular volume
HGB: hemoglobin
MCH: mean corpuscular hemoglobin
MCHC: mean corpuscular hemoglobin concentration
AST: aspartate aminotransferase
ALT: alanine aminotransferase
ALP: alkaline phosphatase
BUN: blood urea nitrogen
CRE: creatinine
GLU: glucose
CHO: total cholesterol
PRO: total protein
CPK: creatine phosphokinase
ALB: albumin
BIL: total bilirubin
yhGM-CSF: yeast-derived hGMCSF
s.c.: subcutaneous
b.wt.: body weight
SD: standard deviation
